# Do ankle brachial index and pulse volume waveforms compare with the Ultrasound Duplex Scan for identifying Peripheral Arterial Disease?

**DOI:** 10.1186/1757-1146-8-S1-A11

**Published:** 2015-04-20

**Authors:** Jane Lewis

**Affiliations:** 1 Cardiff and Vale University Health Board, Cardiff Metropolitan University, UK

## Aims/Objectives

To compare the sensitivity, specificity and overall accuracy of an automated ankle-brachial index (ABI) and pulse volume waveform (PVR) with the Ultrasound Duplex Scan (UDS) for identifying Peripheral Arterial Disease (PAD).

## Content of presentation

200 patients referred for UDS of lower limb arteries at two Medical Physics departments in the UK underwent an automated ABI and PVR measurement using a device utilising volume plethysmography followed by a UD Scan. PAD was recorded for automated ABI if <0.9 (and noted if >1.30), PVR’s if graded mild/moderate/severe and with a haemodynamically significant stenosis or occlusive disease with the UDS. A result of PAD or NO PAD was recorded for each patient and each method. The outcome measure for this study was the agreement and overall accuracy between the automated ABI and UDS results and the PVR and UDS results.

## Relevance/Impact

Of the 200 patients recruited 65% were male, 35% female with an overall mean age of 67 years (range 25-90 (SD 12.38)). 26.7% had DM, 36.7% had CHD, and 28.9% were smokers. 38% were found to have PAD using the gold standard UDS. Those with DM and PAD = 7%, CHD and PAD = 15% and smokers with PAD = 16.4%. The overall results indicated good agreement between ABI and UDS (sensitivity 85%, specificity 89% with overall accuracy 88%) and between PVR and UDS (sensitivity 97%, specificity of 89%, with overall accuracy 95%).

## Discussions

The combined use of the ABI and PVR within one device could enhance vascular assessment especially those with potentially calcified vessels and for treatment planning of leg and foot wounds. With its rapid assessment time, it also has the potential to be introduced into a primary care screening environment as a reliable tool for confirming symptomatic PAD and early identification of asymptomatic PAD.

